# Pristimerin attenuates cell proliferation of uveal melanoma cells by inhibiting insulin‐like growth factor‐1 receptor and its downstream pathways

**DOI:** 10.1111/jcmm.14623

**Published:** 2019-09-11

**Authors:** Xinshu Xie, Saisai Xie, Changying Xie, Yuanying Fang, Zhifeng Li, Rikang Wang, Wei Jiang

**Affiliations:** ^1^ National Pharmaceutical Engineering Center for Solid Preparation in Chinese Herbal Medicine Jiangxi University of Traditional Chinese Medicine Nanchang China; ^2^ Affiliated Hosptial of Jiangxi University of Traditional Chinese Medicine Nanchang China; ^3^ Guangdong Provincial Key Laboratory of New Drug Design and Evaluation, School of Basic Medical Sciences Shenzhen University Health Science Centre Shenzhen China

**Keywords:** Akt, mTOR, pristimerin, IGF‐1, uveal melanoma, ERK1/2

## Abstract

Uveal melanoma (UM) has a high mortality rate due to liver metastasis. The insulin‐like growth factor‐1 receptor (IGF‐1R) is highly expressed in UM and has been shown to be associated with hepatic metastases. Targeting IGF signalling may be considered as a promising approach to inhibit the process of metastatic UM cells. Pristimerin (PRI) has been demonstrated to inhibit the growth of several cancer cells, but its role and underlying mechanisms in the IGF‐1‐induced UM cell proliferation are largely unknown. The present study examined the anti‐proliferative effect of PRI on UM cells and its possible role in IGF‐1R signalling transduction. MTT and clonogenic assays were used to determine the role of PRI in the proliferation of UM cells. Flow cytometry was performed to detect the effect of PRI on the cell cycle distribution of UM cells. Western blotting was carried out to assess the effects of PRI and IGF‐1 on the IGF‐1R phosphorylation and its downstream targets. The results indicated that IGF‐1 promoted the UM cell proliferation and improved the level of IGF‐1R phosphorylation, whereas PRI attenuated the effect of IGF‐1. Interestingly, PRI could not only induce the G1 phase accumulation and reduce the G2 phase induced by IGF‐1, but also could stimulate the expression of p21 and inhibit the expression of cyclin D1. Besides, PRI could attenuate the phosphorylations of Akt, mTOR and ERK1/2 induced by IGF‐1. Furthermore, the molecular docking study also demonstrated that PRI had potential inhibitory effects on IGF‐1R. Taken together, these results indicated that PRI could inhibit the proliferation of UM cells through down‐regulation of phosphorylated IGF‐1R and its downstream signalling.

## 1 INTRODUCTION

Uveal melanoma (UM) is the most common intraocular cancer type in adults.^1^ Although excellent local treatments are currently available, yet no significant progress has been made in changing the disease course. Uveal melanoma mainly metastasize to liver, and the ratio of patients developing liver metastases is approximately 50%.^2^ To date, the underlying reason for the aggressive liver metastasis is still not known, and no treatment has been effective enough to prolong survival. Therefore, it's crucial to look for new molecular targets in order to provide more effective treatments for UM.

Insulin‐like growth factor‐1 is a strong mitogen, which can stimulate IGF‐1R signalling, thereby playing an important role in the occurrence and growth of several cancers.^3^, ^4^, ^5^ Recent studies have shown that the IGF‐1 receptor was highly expressed in UM, and it has been related to tumour prognosis.^6^ insulin‐like growth factor‐1 binds to its receptor, which activates intrinsic receptor tyrosine kinase activity leading to autophosphorylation of IGF‐IR, and subsequently activates the downstream phosphatidylinositol 3′‐kinase (PI3K)‐Akt and mitogen‐activated protein kinases (MAPK) signalling pathways.^7^ In other cells, IGF‐1R activation can mediate the activation of mammalian target of rapamycin (mTOR) both in vitro and in vivo, and the tyrosine phosphorylation of the endogenous mTOR can be induced by IGF‐1 stimulation.^8^ Insulin‐like growth factor‐1 receptor is also associated with the Akt/mTOR signalling activation and the extracellular signal‐regulated kinase 1/2 (ERK1/2) in cancer cells.^9^ However, mutant IGF‐1 receptors with reduced autophosphorylation level show severely impaired mitogenic and tumourigenic activities.^10^ Therefore, identification of the small molecules capable of inhibiting the tyrosine phosphorylation of IGF‐1R subunit is the most effective way to prevent IGF‐1R signalling in cancer cells.

Pristimerin (PRI), a quinonemethide triterpenoid compound, has been widely used as antioxidant, antimalarial, anti‑inflammatory and insecticidal agent.^11^ In recent years, PRI has been shown to inhibit the growth in several cancer cells.^12^, ^13^, ^14^, ^15^ However, the inhibitory effects of PRI on human UM cancer cells and its inhibitory potential in IGF‐1 and IGF‐1R‐mediated tumourigenesis have not yet been investigated. Thus, the present work emphasizes the inhibitory effects of PRI on IGF‐1R and downstream signal transduction.

## 2 MATERIALS AND METHODS

### 2.1 Materials

Pristimerin (purity >99%) was obtained from Chengdu PureChem‐Standard Co., Ltd; IGF‐1, poly‐L‐lysine, bovine serum albumin, 3‐(4,5‐Dimethylthiazol‐2‐yl)‐2,5‐diphenyl‐tetrazolium bromide (MTT) and dimethyl sulfoxide (DMSO) were commercially obtained from Sigma; Antibiotics, Dulbecco's Modified Eagle's Medium (DMEM), trypsin and foetal bovine serum (FBS) were purchased from Gibco‐BRL; Anti‐β‐actin, anti‐phospho‐IGF‐1R (Tyr1135/Tyr1136), anti‐IGF‐1R, anti‐p21^Waf1/Cip1^ (p21) and anti‐Cyclin D1 were from Signalway Antibody. Anti‐phospho‐Akt (Thr308), anti‐ERK1/2, anti‐phospho‐Akt (Ser473), anti‐phospho‐mTOR (Ser338), anti‐Akt and anti‐phospho‐ERK1/2 (Thr202/Tyr204) were from cell signalling technology.

### 2.2 Cell culture

Human uveal melanoma cell lines (UM cells) were obtained from Shanghai Bioleaf Biotech Co., Ltd. And they were maintained in DMEM supplemented with 10% (v/v) FBS, 100 µg/mL streptomycin and 100 U/mL penicillin and incubated at 37°C under 5% CO_2_. The culture medium was changed every 3 days.

### 2.3 MTT assay

Cell viability was determined by MTT assay according to the method described in our previous study.^16^ Briefly, the cells suspended in the medium were seeded in 96‐well plates with a density of 1 × 10^4^ cells/well. After being grown at 37°C in a humidified incubator with 5% CO_2_ for 24 hours, the cells were incubated with PRI or/and IGF‐1 for another 24 hours. After treatment, 10 µL of MTT (5 mg/mL) was added to each well, and the mixture was incubated for 2 hours at 37°C. Then, the MTT reagent was removed, and DMSO (100 μL per well) was added to dissolve the formazan crystals. After shaking the mixture at room temperature for 10 minutes, absorbance was measured at 570 nm using a microplate reader (BioTek Instruments). Results were expressed as the percentage of the absorbance of control cells, which was set at 100%.

### 2.4 Clonogenic assay

Uveal melanoma cells (200/well) were seeded in 6‐well plates for 7 days after the treatment with PRI and/or IGF‐1. After completing the experiment, the cell growth medium was removed, and the cells were washed with phosphate‐buffered saline (PBS). Then they were fixed with 4% paraformaldehyde, and the colonies were stained with crystal violet (0.2%). The colonies of more than 50 cells were counted. All experiments were performed at least three times.

### 2.5 Flow cytometry assay

Flow cytometry was carried out following the protocols routinely used in our laboratory.^17^ Briefly, the cells were seeded into 6‐well plates and treated with IGF‐1 in the presence or absence of PRI for 24 hours. After that, the cells were harvested and washed twice with ice‐cold PBS. Then they were fixed overnight in ice‐cold 70% ethanol and stained with a mixture of Ribonuclease A (RNase A) and propidium iodide (PI). The stained cells were analysed by flow cytometry, and the experiments were repeated three times.

### 2.6 Western blotting analysis

Western blotting analysis was performed as our previously described method.^18^ Cells were lysed with ice‐cold RIPA lysis buffer, and the corresponding protein concentration was determined by a BCA protein assay kit under the manufacturer's instructions. Equal amounts of lysate protein (20 μg/lane) were subjected to sodium dodecyl sulphate polyacrylamide gel electrophoresis (SDS‐PAGE) with 10% polyacrylamide gels and then electrophoretically transferred to nitrocellulose membranes. After transfer, the nitrocellulose blots were blocked with 3% BSA in PBST buffer (PBS with 0.01% Tween 20, *PH* 7.4) and incubated with primary antibodies in PBST containing 1% BSA overnight at 4°C. Immunoreactivity was determined using sequential incubation with horseradish peroxidase‐conjugated secondary antibodies and detected by the enhanced chemiluminescence (ECL) technique.

### 2.7 Molecular docking modelling assay

Molecular modelling studies were carried out by a Molecular Operating Environment (MOE) software version 2015.10 (Chemical Computing Group). The X‐ray crystallographic structure used to establish the template of IGF‐1R kinase (PDB code 5HZN) was downloaded from the Protein Data Bank (PDB). All water molecules in PDB files were deleted, and hydrogen atoms were subsequently added to the protein. The compound PRI was built by the MOE builder module, and energy minimized using the Merck molecular force field MMFF94x with RMSD gradient of 0.05 kcal mol^−1^ Å^−1^. After that, the PRI was docked into the active site of the protein by using the Triangle Matcher method, and the dock scoring in the MOE software was done using the London dG scoring function, and the rigid receptor was taken as the refinement method. After docking, the best five poses of molecules were retained and scored. The geometry of the resulting complex was analysed by the MOE's pose viewer utility.

### 2.8 Statistical analysis

All the results were expressed as means ± SEM (n = 3‐5 times). Analysis of variance (ANOVA) was used to analyse the differences between the groups, followed by the Tukey‐Kramer or Dunnett's multi‐comparison test with Predictive Analytics Software (PASW) (SPSS Inc.). *P* < .05 was regarded as statistically significant.

## 3 RESULTS

### 3.1 PRI suppressed proliferation and colony formation induced by IGF‐1 in UM cells

**Figure 1:**
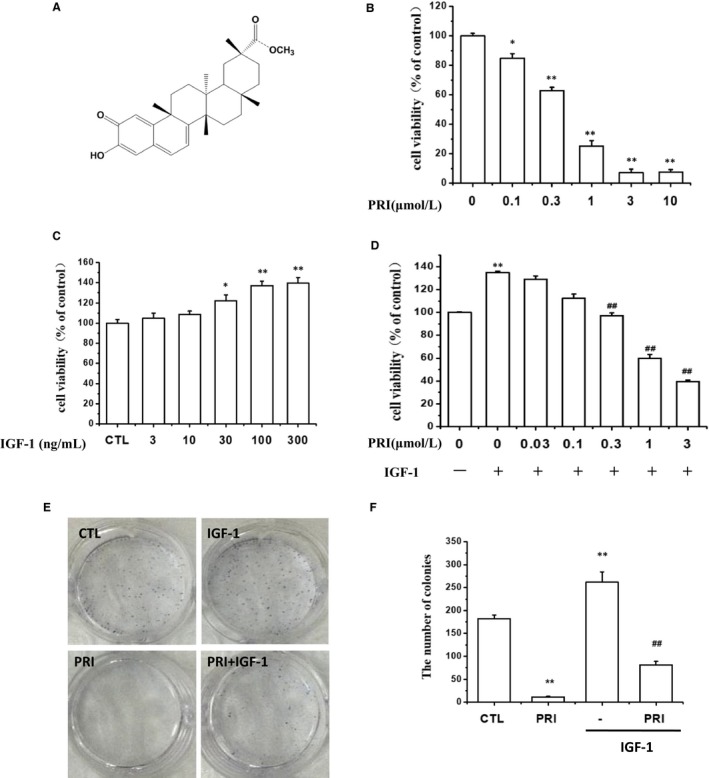
Effects of PRI on proliferation and colony formation of UM cells. A, Chemical structure of PRI. B, UM cells were treated with indicated concentrations of PRI (0‐10 μmol/L) for 24 h, and cell viability was assessed by MTT assay. C, UM cells were treated with various concentrations of IGF‐1 (3‐300 ng/mL) for 24 h, and the cell viability was measured by MTT assay. D, Cells were pre‐treated with various concentrations (0‐3 μmol/L) of PRI for 2 h and then incubated with IGF‐1 for a further 24 h. Cell viability was determined by MTT assay. E, UM cells were seeded in 6‐well plates for 7 days after the treatment of PRI and IGF‐1, fixed with 4% paraformaldehyde and stained with 0.2% crystal violet. F, The statistic results of each colony formation assay. All data are represented as mean ± SD from triplicate wells. *P < .05, **P < .01 vs control, ^##^ *P* < .01 vs IGF‐1‐treated alone group

Figure 1A shows the chemical structure of PRI. The inhibitory activity of PRI on UM cells was investigated by the cell viability assay. As can be seen in Figure 1B, PRI can inhibit cell proliferation in a dose‐dependent manner and significantly reduce the number of cultured live cells. In order to determine the possible effect of IGF‐1 on cancer cell growth, UM cells were first treated with IGF‐1 at different concentrations (3‐300 ng/mL), and the MTT assay was carried out to detect the cell growth. The results indicated that IGF‐1 improved the cell viability in a dose‐dependent manner with the maximum effect at 100 ng/mL (Figure 1C). Thus, this concentration was selected for further experiments. To confirm the inhibitory effect of PRI on cell viability, a colony formation assay was performed. The results from the MTT assay showed that PRI inhibited cell proliferation induced by IGF‐1 in a dose‐dependent manner (Figure 1D) after the cells were seeded in 6‐well plates and colonies were formed for 1 week. As shown in Figure 1E, PRI (1 μmol/L) significantly inhibited colony formation of UM cells and showed a very significant difference in comparison to the control group. These results were in line with the MTT assay. In contrast, IGF‐1 treatment displayed an increased number of colonies, but PRI significantly inhibited colony formation induced by IGF‐1 (Figure 1F). Overall, these results indicated that PRI could inhibit the UM cell proliferation induced by IGF‐1.

### 3.2 PRI regulated the cell cycle distribution of UM cells

**Figure 2:**
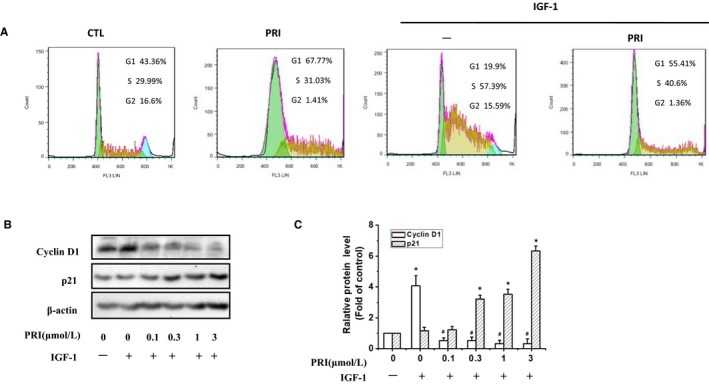
PRI affected the cell cycle progression and the downstream target genes in UM cells. A, UM cells were treated with PRI (1 μmol/L) for 40 min and then were treated with or without IGF‐1 in a serum‐free medium for 40 min. Cells were stained with propidium iodide (PI) and analysed using a flow cytometer. The cell cycle distribution (%) was calculated using FlowJo 7.6. B, Pre‐treatment of UM cells with indicated concentration of PRI for 40 min was followed by treatment with or without 100 ng/mL IGF‐1 for 40 min. The expressions of cyclin D1 and p21 were determined by western blotting. C, Quantification of the immunoblot was performed using densitometric analysis. The results represent prototypical examples of experiments replicated at least three times. *P < .05 vs control groups, ^#^ *P* < .01 vs IGF‐1‐treated alone group

Next, the effect of PRI on the cell cycle distribution of UM cells was examined. After treatment with PRI (1 μmol/L) for 24 hours, the UM cells were processed and stained with propidium iodide (PI). Then, a flow cytometry assay was performed to determine the cell cycle distribution. From Figure 2A, it can be seen that IGF‐1 induced S phase accumulation, but cells in the G1 phase were diminished, and PRI reversed the effect of IGF‐1. Treatment with PRI (1 μmol/L) alone also induced G1 phase accumulation and reduced G2 phase accumulation.

P21 and Cyclin D1 are key regulators in cell cycles. Thus, western blotting was also performed to investigate the involvement of IGF‐1 in regulating the expression of p21 and cyclin D1. It can be seen from the Figure 2B,C) that treatment with IGF‐1 boosted the expression of cyclin D1 but had no effect on p21, while PRI prevented the expression of cyclin D1 and enhanced the expression of p21 in a dose‐dependent manner. This demonstrated that PRI could affect UM cell cycle distribution as well as p21 and cyclin D1 expression.

### 3.3 PRI inhibited IGF‐1‐induced tyrosine phosphorylation of IGF‐1R in UM cells

Previous studies indicated that IGF‐1 could prompt the UM cell proliferation. Accordingly, the signalling pathways responsible for this effect were investigated in present study. We hypothesized that the tyrosine phosphorylation of IGF‐1R stimulated by IGF‐1 is the initial and essential step of IGF‐1 signalling.

**Figure 3:**
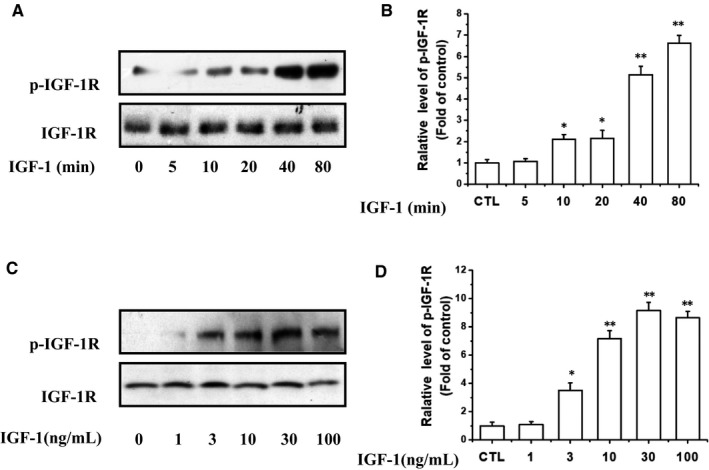
Time‐ and dose‐dependent IGF‐1 activated IGF‐1R. A, UM cells were treated with 100 ng/mL IGF‐1 for scheduled time or C, cells were treated with scheduled concentrations of IGF‐1 for 40 min and the phosphorylation of IGF‐1R was determined by western blotting. B and D, show the densitometric analysis of the immunoblot was expressed as the fold of control. *P < .05, **P < .01 vs control groups. The results represent prototypical examples of experiments replicated at least three times

At the beginning of the experiment, IGF‐1 (100 ng/mL) was used to stimulate the tyrosine phosphorylation of IGF‐1R at different time points from 5 to 80 minutes (Figure 3A,B). The results showed that the phosphorylation levels of these two kinases induced by IGF‐1 were significantly increased within 20 minutes and peaked at 40‐80 minutes. Consistently, the IGF‐1 also induced the phosphorylation of IGF‐1R in a concentration‐dependent manner (Figure 3C,D). The tyrosine phosphorylation of IGF‐1R in UM cells was observed at a concentration of 3 ng/mL of IGF‐1 and increased as the concentration of IGF‐1 increased to a maximum of 30 ng/mL.

**Figure 4:**
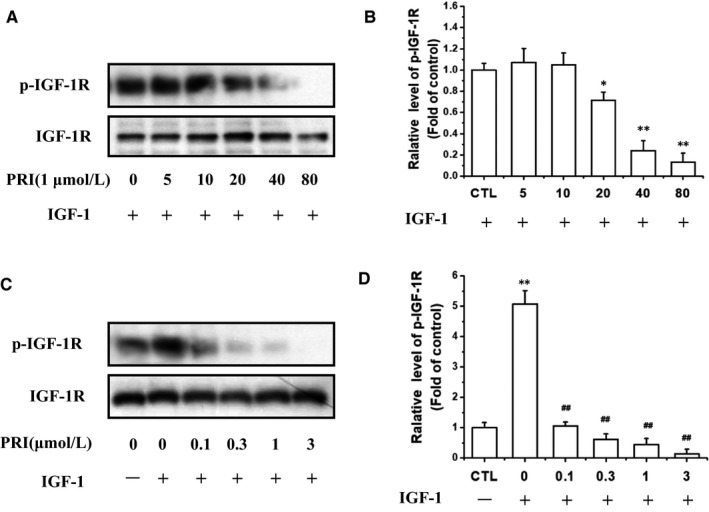
PRI attenuated IGF‐1R activation induced by IGF‐1 in UM cells. A, UM cells were treated with various concentrations of PRI and 100 ng/mL IGF‐1. The levels of p‐IGF‐1R were determined by western blotting. C, UM cells were treated with 1 μmol/L PRI and 100 ng/mL IGF‐1 at various time points. The levels of p‐IGF‐1R were determined by western blotting. B and D, show the densitometric analysis and the p‐IGF‐1R/IGF‐1R ratio was determined. *P < .05 vs control group; ^#^ *P* < .05 vs IGF‐1‐treated alone group. The results represent prototypical examples of experiments replicated at least three times

We then explored whether PRI could inhibit the IGF‐1R activation in UM cells. As shown in Figure 4A,B, after co‐treatment of cells with PRI (1 μmol/L) and IGF‐1 (100 ng/mL) in a serum‐free medium, it can be seen that PRI can reduce the IGF‐1‐induced IGF‐1R phosphorylation in a time‐dependent manner. Furthermore, PRI inhibited IGF‐1‐induced IGF‐1R phosphorylation of Tyr1135/Tyr1136 in a dose‐dependent manner in UM cells. As shown in Figure 4C,D, PRI fully blocked IGF‐1R phosphorylation at 3 μmol/L. Therefore, these results indicated that IGF‐1 led to a rapid phosphorylation of IGF‐1R in UM cells, whereas PRI attenuated the tyrosine phosphorylation of IGF‐1R in a time‐ and concentration‐dependent manner.

### 3.4 PRI suppressed IGF‐1‐induced IGF‐1R signalling pathway

**Figure 5:**
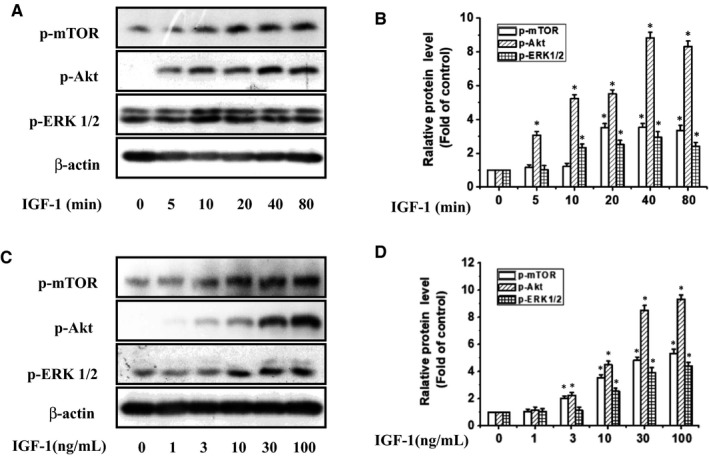
Time‐ and dose‐dependent IGF‐1 increased the phosphorylation levels of mTOR, Akt and ERK1/2 in UM cells. UM cells were treated with IGF‐1 at 100 ng/mL for A, various time points (10‐80 min) and C, various concentrations (0‐100 ng/mL) for 40 min. The phosphorylation of mTOR, Akt and ERK1/2 in UM cells was analysed by western blotting. B and D, Densitometric analysis of the immunoblot was expressed as the fold of control. *P < .05 vs control groups. Results represent prototypical examples of experiments replicated at least three times

As PI3K/Akt/mTOR and MAPK pathways are the main downstream signalling pathways of IGF‐1R, we further determined whether they were participated in the anti‐proliferative action of PRI in UM cells stimulated by IGF‐1. After treating UM cells with IGF‐1 at various time points (0‐80 minutes) or stimulating them with 1‐100 ng/mL of IGF‐1 for 40 minutes, the extent of phosphorylation of mTOR, Akt and ERK1/2 was determined by western blotting. As shown in Figure 5, IGF‐1 stimulated mTOR, Akt and ERK1/2 phosphorylation in a time‐ and dose‐dependent manner.

**Figure 6:**
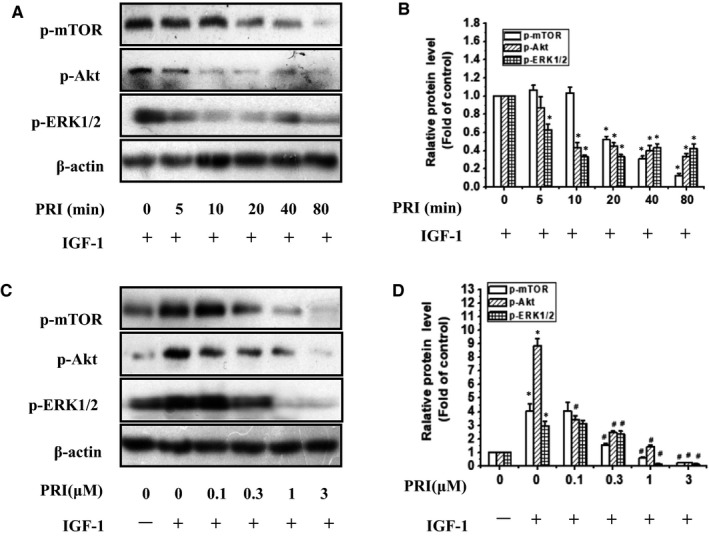
PRI attenuated the phosphorylation levels of mTOR, Akt and ERK1/2 in UM cells in a time‐ and dose‐dependent manner. A, Cells were pre‐treated with PRI at 1 μmol/L for a scheduled time and then incubated with 100 ng/mL IGF‐1 for 40 min. The phosphorylation of mTOR, Akt and ERK1/2 were determined by western blotting. B, Cells were pre‐treated with scheduled concentrations (0‐3 μmol/L) of PRI for 40 min and then incubated with IGF‐1 for 40 min. The phosphorylation of mTOR, Akt and ERK1/2 were determined by western blotting. C and D, Densitometric analysis of the immunoblot was expressed as the fold of control. *P < .05 vs control groups, ^#^ *P* < .05 vs IGF‐1 treated group. Results represent prototypical examples of experiments replicated at least three times

Then the UM cells were treated with 1 μmol/L PRI at various time points (0‐80 minutes) and stimulated with 100 ng/mL of IGF‐1 for 40 minutes. The results indicated that PRI decreased the IGF‐1‐induced mTOR, Akt and ERK1/2 phosphorylation in a time‐dependent manner (Figure 6A,B). Besides, the dose‐course action of PRI was also tested in present work. UM cells were pre‐treated with PRI at various concentrations (0.1‐3 μmol/L) for 40 minutes and then incubated with IGF‐1 (100 ng/mL) for 40 minutes. As shown in Figure 6C,D, PRI could attenuate the activation of mTOR, Akt and ERK1/2 in a dose‐dependent manner. This result was consistent with tyrosine phosphorylation of IGF‐1R induced by IGF‐1. Both Akt and ERK1/2 phosphorylation were significantly blocked at 0.1 μmol/L and 0.3 μmol/L, respectively. Similar results were also observed in the phosphorylation of mTOR, which were blocked at 0.3 μmol/L (Figure 6C,D). These results suggested that PRI could not only inhibit the phosphorylation of IGF‐1R but also could inhibit the IGF‐1R‐mediated signalling pathways.

### 3.5 Identifying PRI as a novel potential IGF‐1R kinase inhibitor

**Figure 7:**
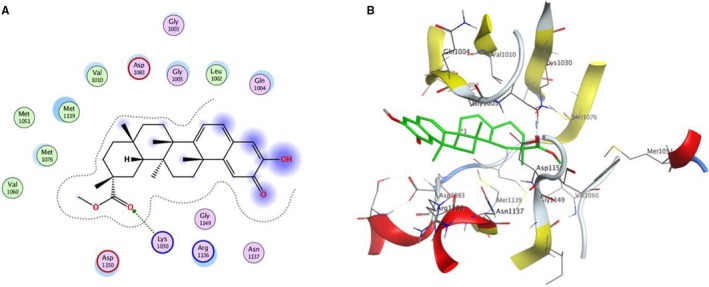
PRI inhibited IGF‐1R kinase. A, The two‐dimensional diagrams displayed the docking model of PRI in active sites of IGF‐1R kinase. D, The three‐dimensional diagram showed the binding conformation of PRI with IGF‐1R kinase. Atom colours: green‐carbon atoms of PRI, grey‐carbon atoms of residues of IGF‐1R kinase, dark blue‐nitrogen atoms and red‐oxygen atoms

In order to validate whether PRI is a novel potential IGF‐1R kinase inhibitor for cancer therapy, a molecular modelling study was carried out using the MOE 2015.10 software package. The X‐ray crystal structure of the IGF‐1R kinase combined with NVP‐AEW541 (PDB code 5HZN) was used to establish the starting model of IGF‐1R. From the Figure 7, it can be seen that PRI fits well in the binding site of IGF‐1R kinase, forming a hydrogen bond between the carbonyl oxygen and Lys 1030. It was also stabilized by Van der Waals and hydrophobic interactions with Gly 1005, Leu 1002, Asp 1150, Gln 1004, Asn 1137, Arg 1136, and Gly 1149. All these results suggested that PRI might function as an IGF‐1R kinase inhibitor and thus impede the signalling pathways mediated by the IGF‐1R kinase.

## 4 DISCUSSION

Insulin‐like growth factor‐1 receptor signalling plays a crucial role in the cell proliferation, migration and cellular invasion of basement membranes. Accordingly, IGF‐1R signalling was regarded as a promising anti‐tumour target, which has been widely studied in clinical trials for different types of tumours including lung cancer, prostate cancer and breast cancer.^5^ In recent years, using either IGF‐1R targeting antibodies or small molecule inhibitors toward the IGF‐1R kinase has been extensively studied.^19^, ^20^, ^21^ High levels of IGF‐1R in UM are closely related to metastatic progression.^22^ Thus, suppression of IGF‐1R provides an effective therapeutic approach to decrease the proliferation of UM cells, especially for cancer cells that have higher expression.

Autophosphorylation and tyrosine kinase activity of IGF‐1R receptors play a crucial role in their signalling functions.^23^Therefore, the improved activity of tyrosine kinases was associated with many cancers and other proliferative diseases.^24^ The signalling pathways and tyrosine kinases in which they participate have thus been identified as promising targets for drug design.^24^, ^25^ In the present study, PRI could significantly reduce the proliferation and the IGF‐1R tyrosine phosphorylation induced by IGF‐1 in UM cells. These findings suggested that PRI targeting IGF‐1R kinases simultaneously resulted in cell cycle arrest and potent anti‐proliferative effects.

Previous studies indicated that the IGF‐1R signalling blocked in vitro by the tyrosine kinase inhibitor could inhibit downstream proliferative signalling through Akt and lead to cell death.^26^, ^27^ In the present study, PRI obviously decreased the tyrosine phosphorylation of IGF‐1R. Moreover, it also inhibited the activation of Akt, mTOR and ERK1/2. In UM tumours, the IGF‐1R/PI3K/Akt pathway is constitutively activated,^28^ and the elevated phosphorylation levels of Akt are related to poor prognosis in most UM.^29^, ^30^ Thus, the IGF‐1R/PI3K/Akt pathway has been regarded as a main target, which is being evaluated as a treatment to further illustrate the importance of targeting activated signalling pathways in different metastatic diseases. Recent studies have shown that mTOR mutations usually occur in melanoma patients and show more negative therapeutic prognosis. Clinical trials with the inhibitors of PI3K/AKT/mTOR pathways may be favourable for melanoma patients with specific mTOR mutations.^31^ In the present work, it was found that IGF‐1 improved the UM cell viability and activated the IGF‐1R/Akt/mTOR and ERK1/2 signalling pathways, while PRI inhibited the UM cell viability and attenuated the IGF‐1R downstream signalling activation. The tumour suppressor protein p21 can regulate the phosphorylation of retinoblastoma and subsequently block of DNA replication to prevent endoreduplication in different cancer cells at both the G1/S and the G2/M cell cycle transitions.^32^ Furthermore, p21, p27^Kip1^ and cyclin D1 play important roles in regulating the cell cycles. P21 serves as a cell cycle progression inhibitor, which can inhibit kinase activity and block progression through G1/S in relation to CDK2 complexes. Our present study found that IGF‐1 stimulated the expression of cyclin D1 in UM cells, while the protein levels of p21 were increased and the levels of cyclin D1 were decreased in UM cells after being treated with PRI. Hence, this result was consistent with the finding that treatment of melanoma with selected signalling kinase inhibitors can effectively decrease proliferation and increase expression of cell cycle inhibitors.^33^ According to the aforementioned molecular docking suggestions, PRI was a potent inhibitor of IGF‐1R, which is consistent with previous reports that compounds with good binding affinity to the IGF‐1R tyrosine kinase have potent activity in the inhibition of cell growth.^20^ All these results confirm the fact that PRI inhibits IGF‐1R and its downstream signalling pathways.

However, future studies including determining whether overexpression of IGF‐1R could rescue the PRI‐induced inhibition of proliferation under the condition that PRI inhibited the activation of IGF‐1R, and investigating the relationship between IGF‐1R inhibition and other signalling pathways in relation to cell growth should be performed to verify. Besides, the IGF‐1R kinase assay should be carried out in the presence of PRI, and an appropriate positive control should also be taken into consideration in these experiments.

In conclusion, our results demonstrated that IGF‐1 stimulated the UM cell proliferation in a dose‐dependent manner, while PRI blocked the role of IGF‐1, and IGF‐1 activated IGF‐1R/Akt/mTOR and ERK1/2 pathways in UM cells. Pristimerin reduced IGF1‐induced IGF‐1R phosphorylation in a dose‐dependent manner. Meanwhile, PRI decreased the expression levels of p‐Akt, p‐mTOR and p‐ERK1/2 in the same manner as p‐IGF‐1R. Accordingly, PRI down‐regulated the oncogenic proteins cyclin D1 and up‐regulated the tumour suppressor p21. The anti‐proliferative effect of PRI in UM cells stimulated with IGF‐1 was mediated by the IGF‐1R/Akt/mTOR and ERK1/2 pathways. Docking simulation by docking PRI into the IGF‐1R active site was carried out to determine the probable binding conformation, and the result indicated that PRI was a novel potent inhibitor of IGF‐1R.

## CONFLICT OF INTEREST

The authors declare no conflicts of interest.

## Data Availability

All data generated or analysed during this study are included in this published paper.
